# Exploring Salivary Biomarkers for Tumor Diagnosis: A Narrative Review

**DOI:** 10.7759/cureus.65725

**Published:** 2024-07-30

**Authors:** Arup Kumar Ghosh, Anurag Nath, Elampavai Elangovan, Abhishek Banerjee, Karthikeyan Ramalingam, Sathya Sethuraman

**Affiliations:** 1 Oral Pathology and Microbiology, Haldia Institute of Dental Sciences and Research, Haldia, IND; 2 Oral Pathology and Microbiology, Sri Venkateshwara Dental College and Hospitals, Bengaluru, IND; 3 Oral and Maxillofacial Pathology, Awadh Dental College and Hospitals, Jamshedpur, IND; 4 Oral Pathology and Microbiology, Saveetha Dental College and Hospitals, Saveetha Institute of Medical and Technical Sciences, Saveetha University, Chennai, IND; 5 Dentistry, Saveetha Dental College and Hospitals, Saveetha Institute of Medical and Technical Sciences, Saveetha University, Chennai, IND

**Keywords:** oral cancers, cancer, saliva, saliva testing, salivary biomarkers, saliva-based diagnostics, proteomics, microvesicles, exosomes, biomarker

## Abstract

A promising method for non-invasive cancer diagnosis and prognosis is through salivary biomarkers. By utilizing the distinct characteristics of saliva and the progress made in biomarker studies, these markers provide more accurate diagnoses for a wider range of malignancies. An attempt was made to thoroughly investigate the field of salivary biomarkers for tumor prognosis and diagnosis, with an emphasis on their use in various cancer forms. Predetermined search criteria were utilized for a systematic search across numerous databases for peer-reviewed papers from 2009 to 2021. Studies concentrating on the detection, validation, and clinical use of salivary biomarkers for different types of cancers were included in the inclusion criteria. Initially, 238 articles were found, of which 15 relevant articles satisfied the inclusion requirements. Information on study aims, methodology, findings, and conclusions were gathered for data extraction.

We identified recurrent themes, patterns, and contradictions by a thematic analysis. We also assessed state-of-the-art salivary biomarkers for tumor diagnosis and prognosis. One major finding is the identification of biomolecules in saliva linked to several cancer forms, including pancreatic, oral, breast, lung, and stomach cancers. There is an increasing amount of evidence demonstrating the value of saliva-based diagnostics in oncology. This is due to new detection methods and developments in salivary proteomics and genomics. Identification of exosomes and microvesicles as salivary biomarker profiles offered molecular understandings of the etiology and evolution of cancer, thereby opening new avenues for diagnosis and treatment. Salivary biomarkers are a non-invasive approach for the early detection and prediction of cancer, thanks to the unique properties of saliva and advancements in biomarker research. This potential revolution could enhance patient outcomes and reduce cancer-related deaths.

## Introduction and background

Early identification and precise prediction are vital to successful treatment planning for treating different types of tumors and greatly impact patient outcomes. Conventional diagnostic methods frequently involve invasive techniques and may not have sufficient sensitivity and specificity for prompt detection. Nevertheless, the development of salivary biomarkers shows potential for improving tumor diagnosis and prognosis [1-6]. Saliva is an easily obtained body fluid and ultrafiltrate that offers numerous benefits as a diagnostic tool [2,7]. In contrast to drawing blood, gathering saliva is painless and patient-friendly. Additionally, saliva contains a variety of biomolecules such as proteins, nucleic acids, metabolites, and exosomes, which indicate both local and systemic physiological changes [2,3,8]. Researchers have been increasingly investigating the potential of saliva as a diagnostic tool for different cancers, utilizing the information it contains. Huang et al. examined the clinical significance and potential future use of biomarkers, specifically outcomes in colorectal cancer [1]. Their study illuminated the important function of biomarkers in influencing clinical decisions and prognosis evaluations for individuals with colorectal cancer. They also emphasized the significance of investigating new methods for diagnosis such as the use of saliva as a diagnostic tool, to improve the early detection and outlook for individuals with colorectal cancer. On the other hand, Lee and colleagues conducted a groundbreaking study that specifically examined saliva-based diagnostics [2]. Their research revealed the effectiveness of saliva as a non-invasive, readily available bodily fluid for diagnostic use.

Lee et al. stressed the importance of saliva as a valuable tool for disease detection and monitoring by showcasing a wide range of salivary biomolecules [2]. Their discovery set the foundation for further research on salivary biomarkers, especially in diagnosing and predicting the outcome of various tumors. By incorporating the knowledge acquired from these studies, we can understand the significance and possibilities of salivary biomarkers in clinical care. These studies highlight the importance of biomarkers in cancer treatment and the potential impact of saliva-based diagnostics in oncology practice. Information about the effectiveness of salivary biomarkers in identifying different kinds of cancers was compiled, and their possible use in early diagnosis, prognosis, and treatment response monitoring was investigated.

Wang et al. reported that salivary analysis of DNA, RNA, proteins, metabolites, and microbiota will be useful in cancer patients [3]. Salivary biomarkers are categorized it into genomic, transcriptomic, proteomic, metabolomic, and microbial types [3-5]. The evaluation also sought to identify knowledge gaps that would benefit from additional research, and highlight significant discoveries, developments, and difficulties in this less explored field. This narrative review aims to describe the current status of salivary research for biomarkers in tumor diagnosis and predicting prognosis.

## Review

Aim

We wanted to comprehensively explore the landscape of salivary biomarkers for tumor diagnosis and prognosis. The present review discusses relevant articles published from 2009 to 2024. Peer-reviewed publications, reviews, and meta-analyses concentrating on salivary biomarkers in tumor diagnosis and prognosis were included in this study. The studies that researched the identification, verification, and clinical use of salivary biomarkers for different tumor types were considered. The review also included research that shed light on the processes, prognostic significance, and diagnostic accuracy of salivary biomarkers. Exclusion criteria included non-English language publications, studies unrelated to salivary biomarkers, tumor diagnosis, or prognosis. Furthermore, the evaluation did not include salivary studies on other oral diseases.

Methods

The search process involved various online databases like PubMed, Scopus, and Web of Science. The search was refined and made more inclusive by using a mix of search terms and Boolean operators like ("salivary biomarkers" OR "saliva-based biomarkers" OR "saliva diagnostics") AND ("tumor diagnosis" OR "cancer detection" OR "prognostic markers"). Furthermore, pertinent MeSH terms were included to improve search accuracy even more. The chosen keywords and search terms included various terminology related to the subject such as “salivary biomarkers”, “diagnosing tumors through saliva”, “markers”, “prognosis”, “detecting cancer”, “saliva proteins”, “genes” “salivary genomics”, “saliva substance”, “genetic”, “salivary tumors”, “oral cancer markers”, “RNA molecules in saliva”. The terms were carefully selected to encompass different studies on salivary biomarkers in oncology, to ensure a comprehensive literature search.

Data extraction entailed gathering relevant information, such as study objectives, methods, important findings, and conclusions, from the selected articles. Between 2009 and 2024, many research studies have helped improve the knowledge and advancement of using salivary biomarkers for diagnosing and predicting tumors, showcasing remarkable progress in this field. However, considering the current review's standpoint and the criteria for inclusion and exclusion, 15 articles were further assessed. A total of 238 articles were identified after the literature search, and 15 pertinent articles were selected to be explained and discussed in this review.

Discussion

Lee et al. started by exploring the possibility of using saliva for diagnostics [2]. Their research highlighted the benefits of utilizing saliva for diagnosis due to its non-invasive properties and the variety of biomolecules that reflect overall health. Loo et al. further examined this topic by comparing human salivary and plasma proteomes [9]. This research gave important information about the proteomic characteristics of saliva in comparison to plasma, emphasizing the distinctive and common biomarkers found in both fluids. Lee et al. conducted salivary diagnostics by directly analyzing the saliva transcriptome [10]. Their study showed that saliva has a lot of gene expression data that could be used for diagnosing diseases by identification of specific RNA signatures. Abraham et al. investigated the potential of using saliva samples instead of blood for high-throughput genotyping [7]. Their research validated the reliability of saliva as a DNA source, expanding the potential of salivary diagnostics to incorporate genetic testing. Wei et al. achieved considerable progress by creating a non-intrusive technique to identify epidermal growth factor receptor (EGFR) gene mutations in lung cancer patients through saliva samples [11]. This research showed that saliva had the potential for cancer diagnostic genetic testing, providing a less invasive alternative to conventional biopsy techniques. Zilberman et al. presented a new microfluidic optoelectronic sensor for detecting gastric cancer in saliva [12]. Their research displayed a new technological method highlighting the importance of medical engineering in improving diagnostic techniques. Huang et al. examined the practical significance and potential future implications of circulating tumor cells and biomarkers in the outcomes of colorectal cancer [1]. This research emphasized the importance of biomarkers in foreseeing clinical results and customizing treatment plans for colorectal cancer.

Rapado-González et al. conducted further research on salivary biomarkers, focusing on cancers located away from the oral cavity [5]. They showed that salivary biomarkers may indicate overall systemic health, thereby expanding the possible uses of saliva-based diagnostics. Majem et al. investigated the function of non-coding RNAs in saliva and recognized them as new biomarkers for molecular diagnostics [8]. Their study emphasized the diagnostic potential of using these non-coding RNAs for detecting different types of cancers in saliva, expanding the range of biomarkers that can be examined. Ghalwash examined the usefulness of salivary biomarkers in diagnosing and predicting outcomes for oral cancer and precancer [13]. This research highlighted the importance of these biomarkers in identifying and predicting the outcome of oral cancers early on, emphasizing the possibility of enhancing patient results with non-invasive tests. A systematic review was conducted by Piyarathne et al. for diagnostic biomarkers in saliva for oral squamous cell carcinoma (OSCC) and oral potentially malignant disorders (OPMD) [14]. They investigated how these biomarkers are linked to different risk factors, presenting a detailed summary of the diagnostic challenges. Skallevold et al. researched salivary biomarkers in lung cancer [15]. Their research helped advance the knowledge of biomarker patterns connected to lung cancer, showcasing the wider usefulness of using saliva tests for diseases beyond oral cancers. Koopaie et al. evaluated salivary biomarkers for diagnosing breast cancer [4]. Their research gathered information regarding the effectiveness of these biomarkers in diagnosis, backing up their possible application in clinical settings for identifying breast cancer. These research projects demonstrated the continuous improvements in salivary biomarkers over the last fifteen years. They highlighted the increasing acknowledgment of saliva's importance as a useful diagnostic fluid, offering vital information for identifying and predicting different types of tumors. Incorporating salivary biomarkers into clinical care shows potential for boosting non-invasive cancer detection, enhancing patient results, and supporting individualized treatment strategies.

**Figure 1:**
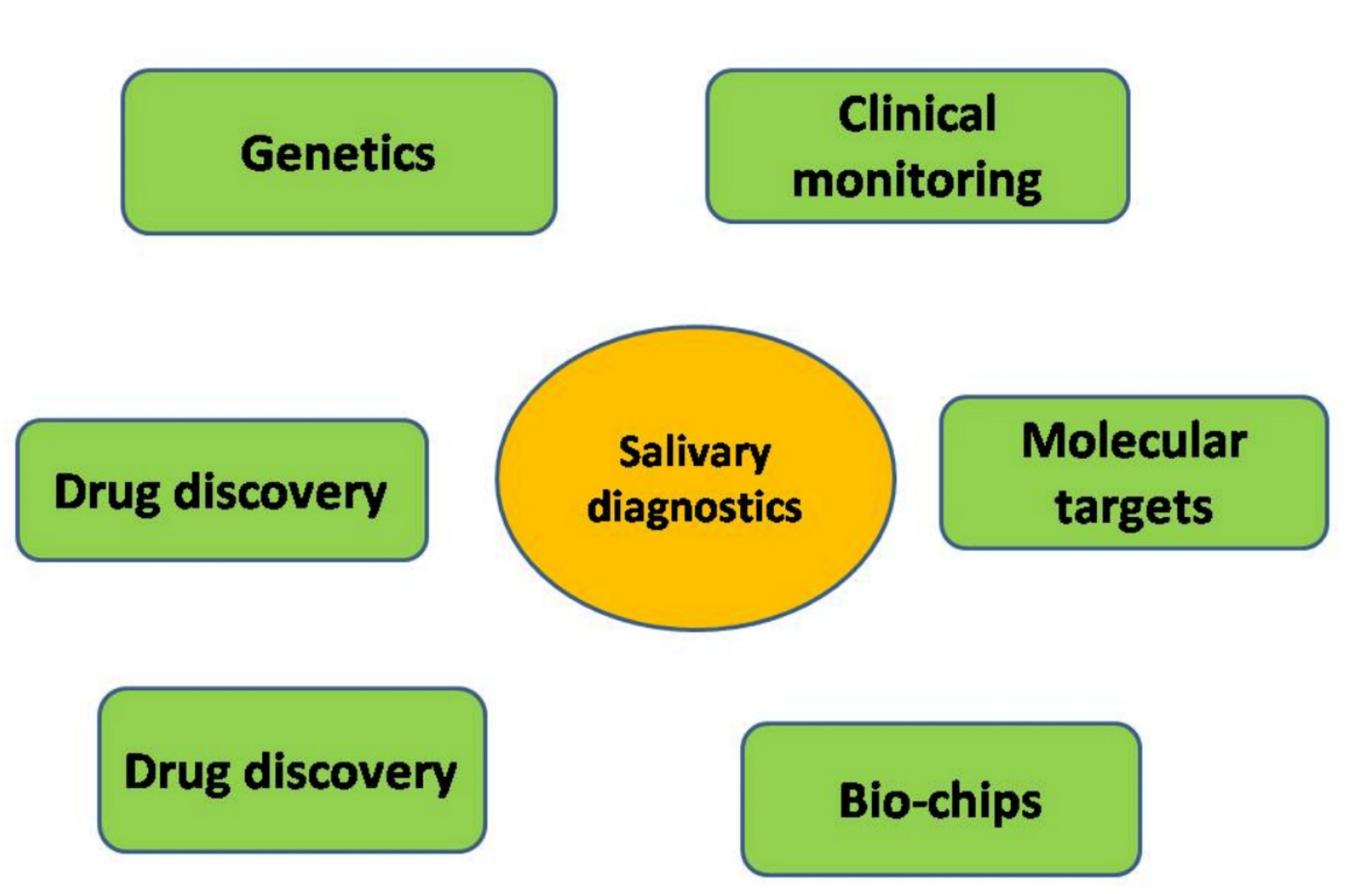
Application of salivary diagnostics encompassing different aspects of the biomedical field

Saliva-based biomarkers, such as proteins, nucleic acids, metabolites, and exosomes, hold significant potential for detecting and predicting the progression of tumors [3,4,8]. These biomarkers in saliva have distinct benefits compared to conventional diagnostic methods, which makes them very attractive for clinical applications. They show disease presence, progression, or response to treatment by reflecting physiological or pathological changes associated with tumors [8]. Saliva is a convenient biological sample that can be easily obtained for diagnostic purposes without invasive procedures. Saliva collection is an uncomplicated, affordable, and patient-friendly process compared to tissue biopsies or venipuncture [7,12]. It presents a low likelihood of infection or harm, making it well-suited for children or older individuals. The diverse biomolecules found in saliva can provide valuable insights into oral health and overall physiological changes, increasing its potential for diagnosis. Various applications of salivary diagnostics are summarized in Figure 1.

Studies have found certain salivary biomarkers associated with different types of cancer such as oral, breast, lung, and colorectal cancers, underscoring saliva's potential as a useful diagnostic tool in oncology [11,15-18]. Conventional tumor diagnostic techniques, such as imaging or tissue biopsies, may not provide the sensitivity or specificity necessary for early identification and can put patients at risk or cause discomfort [3,11,18]. On the other hand, salivary biomarker analysis provides a real-time, non-invasive method with possibly higher sensitivity and specificity [3,11]. It can be a valuable adjunct for patient evaluation and supporting individualized treatment plans [4,15]. With saliva's distinct benefits and the knowledge gained from salivary biomarkers, scientists hope to transform the fields of tumor detection and prognosis.

Salivary Biomarkers in Oral Cancer Detection

Oral cancer poses a significant public health concern worldwide, as it leads to cancerous transformations in the oral mucosal cells of various areas in the mouth such as the lips, tongue, cheeks, floor of the mouth, and hard palate. Detecting oral cancer early is essential for better patient outcomes and lowering mortality rates [6]. López-Jornet et al. and colleagues assessed salivary biomarkers in individuals with OPMD [16]. Their study comparing cases and controls revealed initial findings on how effective these biomarkers are in diagnosing OPMD, hinting at potential directions for further research and practical use. Khijmatgar et al. conducted a thorough examination and network meta-analysis on saliva biomarkers for the early detection of OSCC and head and neck squamous cell carcinoma (HNSCC) [17]. Their research underlined the accuracy of diagnosis and the practicality of using these biomarkers, providing useful information for early detection approaches in these types of cancer. Salivary biomarkers offer a hopeful, non-invasive method for identifying and tracking oral cancer, giving an understanding of disease development and advancement. Increased amounts of certain proteins, like matrix metalloproteinases (MMPs), vascular endothelial growth factor (VEGF), interleukins (ILs), and cancer-associated antigens, have been associated with the development of oral cancer. MMP-9 is an enzyme related to modifying the extracellular matrix. It can be a possible saliva biomarker for oral cancer because of its heightened presence in patients with OSCC [19]. Moreover, changes in the levels of certain microRNAs (miRNAs) found in saliva, including miR-21, miR-31, miR-181a, and miR-196a, have been linked to oral cancer progression [8]. Novel saliva biomarkers have been recently discovered, and new detection techniques for oral cancer have been created. Chandrasekaran and colleagues examined proteins and nucleic acids in saliva to identify oral cancer, emphasizing their possible use in diagnosis. Guillon and colleagues reviewed the utilization of salivary samples in oral cancer diagnosis, highlighting the importance of different biomarkers [20]. Bastías et al. investigated how MMPs, cytokines, and exosomes contribute to the detection of oral cancer, offering a better understanding of the disease's molecular mechanisms [21]. Utilizing these biomarkers found in saliva can enhance early detection methods, improve patient results, and lessen the worldwide impact of oral cancer.

Salivary Markers in Systemic Tumors

Salivary biomarkers are becoming increasingly important for the early detection and prognosis of a range of systemic cancers such as breast, lung, pancreatic, and gastric cancer. These biomarkers provide a non-invasive and easily accessible way to detect cancer, offering chances to enhance patient outcomes with timely intervention and personalized treatment methods. Research has explored the effectiveness of salivary biomarkers in detecting breast cancer, yielding encouraging findings. Koopaie M et al. performed a systematic review and meta-analysis, uncovering the diagnostic value of salivary biomarkers in breast cancer [4]. More specifically, researchers have linked the salivary protein CA15-3 with the advancement of breast cancer, offering valuable information for monitoring the disease and predicting outcomes. Progress in the field of salivary proteomics has allowed for the discovery of new biomarkers, improving the precision of diagnoses and personalized approaches to treatment. Non-invasive saliva tests for lung cancer can identify cancer-related mutations like epidermal growth factor receptor (EGFR) gene mutations. Wei et al. showed that it is possible to identify EGFR mutations in saliva samples, highlighting the promise of using salivary biomarkers for non-invasive molecular analysis in individuals with lung cancer [11]. Skallevold et al. explored salivary biomarkers associated with inflammation in lung cancer, offering valuable knowledge on the underlying mechanisms of the disease [15].

Salivary biomarkers have displayed potential in the early identification and prediction of pancreatic cancer, which is known for being diagnosed in advanced stages and having low survival rates. A group of mRNA biomarkers in saliva were identified that could accurately distinguish between pancreatic cancer patients and those without cancer with high levels of sensitivity and specificity [3]. Progress in analyzing salivary miRNA has resulted in finding variations in miRNA levels in pancreatic cancer patients, which could serve as potential indicators for detecting and tracking the disease [19]. Promising salivary biomarkers have been discovered for diagnosing and predicting the prognosis of gastric cancer. Zilberman et al. created a tiny optoelectronic sensor that can detect salivary metabolites linked to gastric cancer, offering a non-intrusive method for screening the disease [12]. The discovery of salivary proteins associated with gastric cancer underscored their promise as potential early-detection biomarkers [15]. In addition to these cancers, researchers have also studied salivary biomarkers in other types of cancers, including colorectal cancer and oral squamous cell carcinoma. Research has shown that these biomarkers have diagnostic and prognostic significance, providing non-invasive methods for detecting and monitoring diseases [1,18]. Investigating salivary biomarkers in systemic malignancies could improve prognosis, early identification, and individualized treatment plans. Better patient outcomes and a decline in cancer-related mortality are possible with this strategy.

Mechanistic Insights (Exosomes and Microvesicles)

Role of exosomes and microvesicles in tumor-derived biomarkers:* *Extracellular vesicles generated by a variety of cell types, including tumor cells, known as exosomes and microvesicles are essential for macromolecules like proteins, lipids, and nucleic acids to diffuse throughout cells and facilitate intercellular communication. These vesicles are becoming recognized as important tumor-derived biomarker mediators, providing important new information about the etiology and course of cancer [14]. Majem et al. clarified the function of exosome-derived non-coding RNAs (ncRNAs) as putative biomarkers in saliva, emphasizing their important role in cancer diagnosis and prognosis [8]. Furthermore, Lee YH et al. (2011) showed that saliva-derived exosomes contained tumor-specific RNA transcripts, highlighting their value as non-invasive biomarkers for cancer diagnosis [10].

Mechanisms of exosome secretion and transport: Exosomes and microvesicles are secreted by tumor cells through a variety of channels, including ceramide-dependent pathways and the endosomal sorting complex needed for transport (ESCRT) machinery [14]. Tumor-derived biomarkers can spread more easily when these vesicles are discharged into the extracellular space and are carried by body fluids like saliva to distant locations. Exosome transport pathways are crucial for cancer progression and demonstrated the role of exosome-associated proteins in mediating their uptake by recipient cells [5].

Impact of Tumor-Derived Exosomes on Salivary Biomarker Profiles

Exosomes produced from tumors can impact salivary biomarker profiles. They are involved in the deregulating molecular pathways linked to the initiation and spread of cancer [14]. Huang et al. showed that tumor-derived exosomes might modify the salivary proteome, indicating that these vesicles regulate host-tumor interactions [1]. Moreover, it has been demonstrated that genetic changes specific to tumors in saliva-derived exosomes offer a valuable understanding of the molecular terrain of cancer and its possible diagnostic consequences [21]. Marakala performed a systematic review and meta-analysis of head and neck cancer biomarkers [22]. Sensitivity was reported to be 29.5% to 100% and specificity was 57.1% to 100%. Combined biomarkers were more effective than individual entities. Kang et al. performed a systematic review and meta-analysis of salivary miRNA in head and neck cancer [23]. They have reported a pooled sensitivity of 69.7% and a specificity of 86.8%. Umapathy et al. discussed salivary proteomics in their review and emphasized the role of salivary biomarkers in the early diagnosis, treatment follow-up, and high-risk monitoring of cancer patients [24]. Barros et al. discussed optimizing salivary sample handling and standardization of evaluatory procedures [25]. Sethuraman et al. elaborated on nanoparticle biosensors and their relevance in salivary detection [26]. Wan et al. analyzed the salivary P90 biomarker in oral leukoplakia [27]. 

Table 1 summarizes the relevant findings observed in this review.

**Table 1:** Summary of the relevant findings

S.No	Authors	Year	Assessed parameters	Detection	Advantage
1.	Huang et al. [1]	2016	Circulating tumor cells	Colorectal cancer	Early detection, customized treatment plans
2	Lee et al. [2]	2009	Saliva transcriptome	Diagnosing diseases by identifying specific RNA signatures.	Non-invasive, readily available
3	Loo et al. [9]	2010	Proteomic characteristics of saliva in comparison to plasma	Gene ontology distributions were similar	Non-invasive
4	Abraham et al. [7]	2012	Reliability of saliva as a DNA source	Salivary diagnostics	Potential genetic testing
5	Wei et al. [11]	2014	Non-intrusive technique to identify epidermal growth factor receptor (EGFR)	Lung cancer	Less invasive alternative to conventional biopsy techniques
6	Zilberman et al. [12]	2015	Microfluidic optoelectronic sensor	Stomach cancer	Improved diagnostic techniques
7	Rapado-González et al. [5]	2020	Meta-analysis	Cancers located far away from the oral cavity	indicator of overall health
8	Majem et al. [8]	2015	Non-coding RNAs	Diagnosing different types of cancer	expansion of the diagnostic range
9	Ghalwash [13]	2020	Review	Oral cancer and precancer	predicting the early outcome of cancer
10	Piyarathne et al. [14]	2021	Systematic review	Oral, potentially malignant disorders	Link between different risk factors
11	Skallevold et al. [15]	2021	Biomarker patterns	Lung cancer	Widening the spectrum
12	Koopaie et al. [4]	2022	Comprehensive evaluation	Breast cancer	Application in clinical settings
13	López-Jornet et al. [16]	2023	Comparing cases and controls	Oral potentially malignant disorders (OPMD)	Potential directions for practical use
14	Khijmatgar et al. [17]	2024	Network meta-analysis	Oral squamous cell carcinoma (OSCC) head and neck squamous cell carcinoma (HNSCC)	Accuracy of diagnosis and practicality, enhancing patient results, and individualized treatment strategies

A promising non-invasive technique for the early diagnosis and prognosis of several malignancies, including pancreatic, stomach, lung, breast, and oral cancers, was made possible by salivary biomarkers. Saliva's special qualities and developments in biomarker research have improved diagnostic precision and individualized therapy options, which may improve patient outcomes and lower the death rate from cancer.

## Conclusions

Salivary biomarkers hold significant promise for non-invasive early detection and prognostication of various cancers, including oral, breast, lung, pancreatic, and gastric cancer. Their benefits lie in the ease and cost-effectiveness of saliva collection, enhanced patient satisfaction, and reduced risk of complications compared to traditional methods like tissue biopsies and blood draws. Advances in biomarker research, particularly the study of exosomes and microvesicles, have improved diagnostic accuracy and personalized treatment, potentially leading to better patient outcomes and reduced cancer mortality. However, challenges remain such as variability in biomarker levels, potential contamination, and the need for standardized protocols to ensure reliability and accuracy. Reviews like this are essential for consolidating current knowledge, identifying gaps, and guiding future research. Clinically, salivary biomarkers could revolutionize cancer diagnosis and treatment by providing a non-invasive, real-time monitoring tool that complements existing methods. Future directions include extensive validation of these biomarkers, developing standardized collection and analysis procedures, and integrating salivary diagnostics into routine clinical practice. Interdisciplinary collaboration, investment in advanced technologies, and rigorous clinical trials will be crucial for fully realizing the potential of salivary biomarkers in oncology.
